# Evaluation of Anatomoradiological Findings on Trigeminal Neuralgia Patients Using Computed Tomography and Cone-Beam Computed Tomography

**DOI:** 10.3390/diagnostics12010073

**Published:** 2021-12-29

**Authors:** Seçil Aksoy, Arzu Sayın Şakul, Durmuş İlker Görür, Bayram Ufuk Şakul, Kaan Orhan

**Affiliations:** 1 Department of Dentomaxillofacial Radiology, Faculty of Dentistry, Near East University, Mersin 10, Lefkosa 99138, Turkey; 2 Department of Medical Pharmacology, School of Medicine, Istanbul Medipol University, Istanbul 34810, Turkey; aasakul@medipol.edu.tr; 3 Department of Oral and Maxillofacial Surgery, Dentiron Private Clinic, Ankara 06690, Turkey; ilkergorur@gmail.com; 4 Department of Anatomy, School of Medicine, Istanbul Medipol University, Istanbul 34810, Turkey; usakul@medipol.edu.tr; 5 Department of Dentomaxillofacial Radiology, Faculty of Dentistry, Ankara University, Ankara 06100, Turkey; call53@yahoo.com; 6 Medical Design Application and Research Center (MEDITAM), Ankara University, Ankara 06100, Turkey; 7 Department of Dental and Maxillofacial Radiodiagnostics, Medical University of Lublin, 20-081 Lublin, Poland

**Keywords:** trigeminal neuralgia, CBCT, CT, foramen ovale, foramen rotundum, impressio trigeminale

## Abstract

The study aimed to establish and evaluate anatomoradiological landmarks in trigeminal neuralgia patients using computed tomography (CT) and cone-beam CT. CT images of 40 trigeminal neuralgia (TN) and 40 healthy individuals were retrospectively analyzed and enrolled in the study. The width and length of the foramen rotundum (FR), foramen ovale (FO), foramen supraorbitale, and infraorbitale were measured. The distances between these foramen, between these foramen to the median plane, and between the superior orbital fissure, FO, and FR to clinoid processes were also measured bilaterally. Variations were evaluated according to groups. Significant differences were found for width and length of the foramen ovale, length of the foramen supraorbitale, and infraorbitale between TN and control subjects (*p* < 0.05). On both sides, FO gets narrower and the length of the infraorbital and supraorbital foramen shortens in the TN group. In most of the control patients, the plane which passes through the infraorbital and supraorbital foramen intersects with impression trigeminale; 70% on the right-side, and 67% in the left-side TN groups. This plane does not intersect with impression trigeminale and deviates in certain degrees. The determination of specific landmarks allows customization to individual patient anatomy and may help the surgeon achieve a more selective effect with a variety of percutaneous procedures in trigeminal neuralgia patients.

## 1. Introduction

The fifth cranial nerve (CN V), also known as the trigeminal nerve, has its origins in the midlateral surface of the pons, near the middle cerebellar peduncle, and comprises a small brachial motor root (portio minor) and a large general sensory root (portio major) [1,2]. It travels anterolaterally, passes below the tentorium cerebelli, and then pierces the dura mater to enter Meckel’s cave in the superior aspect of the petrous temporal bone of the middle cranial fossa. Meckel’s cave can be described as a dural recess and contains the cerebrospinal fluid between the two layers of the dura mater. It houses trigeminal ganglion, and its open-ended three-fingered glove shape provides a canal for all three major branches of the trigeminal nerve to supply sensory and motor innervation. The fingers of the glove contain the ophthalmic (V1), maxillary (V2), and mandibular (V3) branches that pass through the superior orbital fissure (FOS), foramen rotundum (FR), and foramen ovale (FO) from superior to inferior, respectively [3,4].

A variety of pathologies may influence the trigeminal nerve, including benign and malign tumors, inflammation, infection, and neuralgias. Trigeminal neuralgia (TN), also called tic douloureux, presents with severe, sudden, unilateral, paroxysmal, and electric-shock-like pain in the area of distribution of one or more divisions of the trigeminal nerve [2]. The precise etiology of trigeminal neuralgia has not been defined, but the opinion most generally held is that the neuralgia is due to the vascular loop compression or contact or compressions due to the tumor, vascular malformation, or cyst. Other reasons for the TN include multiple sclerosis, virus infections, and narrow foramen ovale and foramen rotundum [5,6].

The imaging techniques for evaluating the trigeminal nerve and its roots in trigeminal neuropathy include magnetic resonance imaging (MRI), computed tomography (CT), cone-beam computed tomography (CBCT), and plain radiography. TN may be produced by a lesion of entire projections of the trigeminal nerve, which may occur in the brainstem segment, cisternal segment, Meckel’s cave, cavernous segment, and extracranial segment. MRI is the preferred imaging modality for evaluating the pathologies involving all segments; however, CT, CBCT, and plain radiographs may also be used in lesions involving the peripheral segment [7].

Conservative medical treatment is the first option to treat trigeminal neuralgia non-invasively. Percutaneous radiofrequency rhizotomy (RF-TR), microvascular decompression, balloon compression, and glycerol rhizolysis may be applied for the patients refractory to medication [2,8]. RF-TR is targeted to establish a thermolesion in the trigeminal root, and intimate knowledge of trigeminal nerve anatomy is crucial to avoid its inadvertent injury to surrounding vital structures during operation. RF-TR may be a difficult procedure, and correct positioning of the electrode is of prime importance for the effectiveness and selectivity of percutaneous trigeminal radiofrequency thermorhizotomy, and the wrong position may cause severe complications such as carotid injuries, intracranial hemorrhage, cranial nerve injuries, and carotid–cavernous fistulae [9].

Thus, this study aimed to establish some anatomoradiological landmarks in trigeminal neuralgia patients using CT and CBCT.

## 2. Materials and Methods

Using retrospective data from our faculty, a power analysis (Power and Precision software, Biostat, Englewood, NJ, USA) was conducted that indicated that detection of differences between patients with and without trigeminal neuralgia could be obtained with 38 patients at a power of 0.8 (alpha = 0.05). Thus, this study was conducted using 40 trigeminal neuralgia patients (15 male, 25 female) (V2 and V3) and 40 healthy individuals (15 male, 25 female) were retrospectively randomly selected for good-quality CT and CBCT images.

In this study, a total of 80 patients’ CT and CBCT images were used. In total, 40 trigeminal neuralgia patients (15 male, 25 female) (V2 and V3) and 40 healthy individuals (15 male, 25 female) were retrospectively analyzed and enrolled in the study. All of the participants signed the consent form before imaging. The study protocol was conducted according to the principles described in the Declaration of Helsinki, including all amendments and revisions, and approved by the Health Sciences Ethics Committee of the University (permit no: YDU/2020/82–1149). Patients with a history of surgery in the midline skull base or trauma were excluded.

CBCT images were acquired with the Netwom 3G (QR Verona, Italy). As a protocol in our clinics, all patients were cooperative and lay in a horizontal position without any movement during all CBCT scans. A 12 inch field of view imaging protocol was used to capture the facial and skull base anatomy. CBCT images were obtained under the following exposure parameters: 120 kVp tube voltage, 3–5 mA tube current, and 0.3 mm axial images with isotropic voxels. Besides, CT images were performed using various CT scanners (Siemens Somaton Sensation, 16 slices, Siemens Medical Solutions, Erlangen, Germany; GE Lightspeed 16 slice, GE Medical Systems, Milwaukee, WI, USA) at 120 kVp and 25 mA with a display matrix of 512 × 512.

The resultant axial images were exported and stored as 512 × 512 matrices in Digital Imaging and Communications in Medicine (DICOM) format and then were imported in Maxilim^®^ software version 2.3.0. (Medicim, Sint-Niklass, Belgium) to generate a good quality 3D image of the patients. The design of the study is very similar to that is used by Oz et al. [10]. Unlike these studies, two planes passing through the supraorbital and infraorbital foramen on both sides were drawn to determine the relation of the impressio trigeminale to the plane.

**Figure 1:**
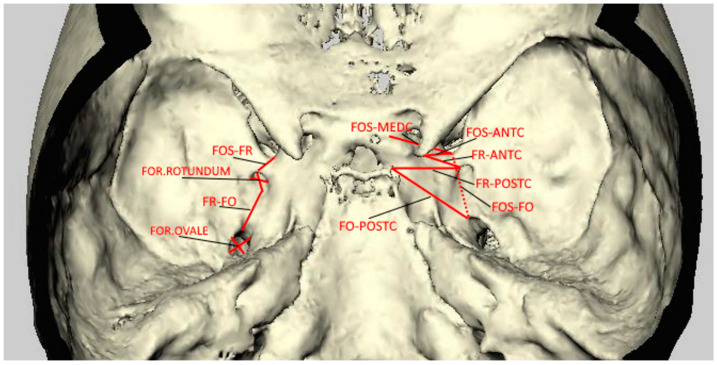
3D representation of the CT image showing all intracranial measurements.

All segmentation and measurements were done on the 3D surface models by the same observer (SA) individually, who was blinded to the clinical features of the patients. After segmentation, measurements of the 3D images were identified and marked by using a cursor-driven pointer on the 3D-surface-rendered volumetric image. The width and length of the FO, FR, and foramen supraorbitale and infraorbitale were measured. The distances between these foramens, between these foramens to the median plane, and between the FOS, FO, and FR to the anterior, middle, and posterior clinoid processes were also measured. All measurements were performed bilaterally (Figure 1). Variations were evaluated according to groups.

Statistical Methods Statistical analyses were carried out using the SPSS 19.0.1 software (SPSS, Chicago, IL, USA). Pearson’s chi square test and Student’s t-test were performed for statistical analysis of differences in with and without TN patients, localizations, and measurements (*p* < 0.05).

## 3. Results

In this study, the overall mean age for trigeminal neuralgia patients was 43.8 (range 27 to 72 years) and for control, the subject was 39.1 years (range 21 to 65 years). It was found that trigeminal neuralgia occurs commonly with advancing age. Thirty-nine (97.5%) had unilateral TN, bilateral involvement occurred in only one (2.5%) of the study group with nearly equal prevalence on both sides (20 TN occurred on the left and 21 TN occurred on the right side).

**Figure 2:**
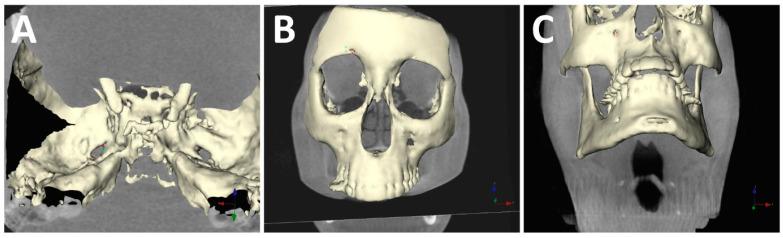
3D measurements of (**A**) foramen ovale; (**B**) foramen supraorbitale; (**C**) foramen infraorbitale.

Table 1 shows the mean values of morphometric analysis. Significant differences were found for the width and length of the foramen ovale, the length of the foramen supraorbitale, and the infraorbitale between TN and control subjects (*p* < 0.05). On both sides, the foramen ovale gets narrower and the length of the supraorbital and infraorbital foramen shortens in the TN group (Figure 2).

The distances between the right and left supraorbital foramen ranged from 41.3 to 60.9 mm with a mean of 49.6 mm in TN patients and ranged from 42.8 to 59.3 mm with a mean of 51.8 mm in control groups. The distances between the right and left infraorbital foramen ranged from 41.4 to 58.1 mm with a mean of 49.3 mm in TN patients and ranged from 42.0 to 60.7 mm with a mean of 51.1 mm in control groups. Distances did not significantly differ between the groups. The mean length of the line from the center of the right infraorbital foramen to the center of the supraorbital foramen was 45.1 and 40.2 mm in TN patients and control subjects, respectively. The mean length of the line from the center of the left infraorbital foramen to the center of the supraorbital foramen was 45.4 and 41.6 mm in TN patients and control subjects, respectively. No statistically significant differences were found between the compared groups.

Table 2 shows the mean distances from the center of the infraorbital foramen, supraorbital foramen, and impressio trigeminale to the median plane. Distances did not significantly differ between the groups.

**Figure 3:**
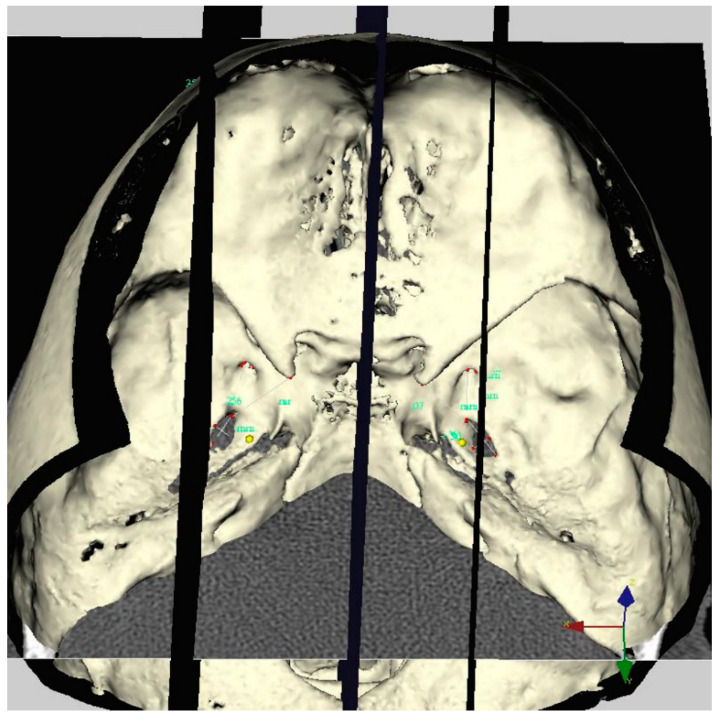
Three-dimensional representation of a TN patient’s skull. No intersection was found on the left side between the supraorbital-infraorbital foramen and impressio trigeminale.

On each side, two planes were drawn passing through the supra and infraorbital foramen and perpendicular to the horizontal plane. The position of the impression trigeminale was evaluated according to this plane. In most of the control patients, this plane intersects with impression trigeminale (70% on the right and 65% on the left side), 70% in the right and 67% in the left-side TN groups. This plane did not intersect with impression trigeminale and deviates in certain degrees (Figure 3).

Examination of the trigeminal neuralgia patients revealed that there was more distance between FOS-FR and FOS-FO than control patients (Table 3). Besides, the distance between the FOS-posterior clinoid and FR-posterior clinoid is higher than in normal patients (Table 4).

## 4. Discussion

The overall incidence of trigeminal neuralgia ranges from 40–50 cases per one million people. Trigeminal neuralgia occurs in females almost twice as often as males. It is a common cause of facial pain and its exact reason still remains unknown [11]. Conservative medical treatment is widely used to relieve trigeminal neuralgia’s sudden, sharp, and paroxysmal pain. Some invasive (microvascular decompression of trigeminal nerve, partial sensory rhizotomy, internal neurolysis, cryotherapy) and non-invasive procedures (stereotactic radiosurgery and low-level laser therapy) are performed for medical treatment failures [12].

Many hypotheses and ideas have been brought forward to clarify the development of TN. Although vascular compression may cause idiopathic TN, this compression may also be found in asymptomatic patients. Neto et al. [6] hypothesized that the higher incidence of the TN on the right side might be due to the asymmetric developments of the foramen, which is narrower on the right side. Bangash [13] also reported 64% of the TN occurred on the right side. Our results showed that trigeminal neuralgia does not differ between sides. On the contrary, Capel and Peltier [14] incriminate arterial or vascular compression by the superior cerebellar artery, the vein of Dandy, or the transverse pontine vein.

Studies were focused specifically on the size of the foramen rotundum and foramen ovale in TN patients. A previous study indicated that the mean width and length of the FO and FR did not significantly differ between the TN patients and healthy subjects [15]. They reported that the average size of the left- and right-side foramen ovale was 4.9 × 5.5 and 4.8 × 6.04 mm in TN patients and 4.1 × 7.6 and 3.7 × 8.2 mm in the control group without any significant differences. They also found the average size of the left and right-side foramen rotundum was 2.8 × 2.9 and 3.04 × 3.2 mm in TN patients and 2.5 × 3.1 and 2.4 × 3.2 mm in control groups. Another study conducted by Liu et al. [16] found that the mean measurements did not significantly differ between the TN and volunteer groups in terms of foramen sizes, but a significant difference was found in the aspect ratio of the foramen ovale between the painful and non-painful side in the TN group. Our results disagree with the previous study in terms of foramen ovale measurements. These differences may be due to the measurement techniques that previous studies used in cross-sectional CT images. On the contrary, a 3D surface-rendering program was used in the present study. However, our TN group measurements between the pain and non-pain sides were also significantly different whereas non-pain sides had similar measurements to the control group.

In the present study, the supraorbital foramen length measuring approximately 2.6 mm in the TN group and 3.6 mm in control patients were determined, similar to the other studies, where the height of the foramen was found between 2.5 and 3.5 mm [17,18,19]. Therefore, reduced vertical diameter might also play a role in trigeminal neuralgia but further studies are needed to understand the mechanism.

Regarding the infraorbital measurements, Nanayakkara et al. [20] stated that the width and length of the infraorbital foramen were 3.27 and 3.11 mm, and 3.33 and 3.31 mm on the right and left side, respectively. Orhan et al. [21] defined the same measurements as 3.52 and 4.84 mm and 3.66 and 4.82 mm on both sides. These previous study results are very similar to measurements in our control group.

According to the results of this study, of 68.4% of sides with trigeminal neuralgia, the supraorbitale-infraorbitale plane did not intersect with impressio trigeminale. However, in 75.4% of healthy sides, supraorbitale–infraorbitale planes intersect with impressio trigeminale. Due to the lack of a previous study with regards to the estimation of the prevalence of this condition, there are no available data in the literature to make a comparison with our results.

The limitation of this study was the absence of soft tissue assessment. The present study compared only hard tissue changes in TN patients and normal subjects, therefore soft tissue evaluations are also needed in further studies.

To the best of our knowledge, the present study is the first to measure and compare the distances between the FO, FR, FOS, and these foramen to clinoid processes in trigeminal neuralgia patients and healthy individuals. These foramen are of great surgical and diagnostic importance in procedures such as percutaneous trigeminal rhizotomy for trigeminal neuralgia, transfacial fine needle aspiration technique in the perineural spread of the tumor, and electroencephalographic analysis for seizures.

As a consequence, the determination of specific landmarks allows customization to individual patient anatomy and may help the surgeon achieve a more selective effect with a variety of percutaneous procedures.

## Figures and Tables

**Table 1:** The dimensions of the foramen ovale, rotundum, supraorbitale and infraorbitale comparison in trigeminal neuralgia patients and control subjects. The same letters indicate statistical significance, which is less than 0.05.

Foramen Ovale	TN Patients				Control Subjects				*p*-Value
	LOW	LOL	ROW	ROL	LOW	LOL	ROW	ROL	
	*n* = 20	*n* = 20	*n* = 21	*n* = 21	*n* = 40	*n* = 40	*n* = 40	*n* = 40	
Mean	**4.8 ^a^**	**6.6 ^b^**	**4.7 ^c^**	**6.3 ^d^**	**5.8 ^a^**	**7.8 ^b^**	**5.8 ^c^**	**7.9 ^d^**	**<0.05**
SD	±0.66	±0.79	±1.20	±1.14	±0.65	±0.76	±0.77	±1.03	
Min	3.8	5.8	2.5	4.0	4.7	6.4	4.5	6.1	
Max	6.1	8.3	6.9	7.8	7.1	9.0	7.2	10.6	
**Foramen** **Rotundum**	**LRW**	**LRL**	**RRW**	**RRL**	**LRW**	**LRL**	**RRW**	**RRL**	
Mean	3.0	3.4	3.1	3.2	3.0	3.7	3.2	3.7	>0.05
SD	±0.29	±0.55	±0.62	±0.70	±0.53	±0.5	±0.49	±0.42	
Min	2.6	2.3	2.5	2.0	2.2	2.8	2.4	2.9	
Max	3.4	3.7	4.2	4.2	4.5	4.8	4.5	4.4	
**Foramen** **Supraorbitale**	**LSOW**	**LSOL**	**RSOW**	**RSOL**	**LSOW**	**LSOL**	**RSOW**	**RSOL**	
Mean	3.1	**2.4 ^e^**	3.1	**2.4 ^f^**	2.9	**3.6 ^e^**	2.8	**3.6 ^f^**	**<0.05**
SD	±0.88	±0.55	±0.68	±0.48	±0.46	±0.58	±0.42	±0.44	
Min	2.4	1.6	2.1	1.6	2.3	2.5	2.2	2.7	
Max	4.6	3.3	4.4	3.5	4.0	4.7	3.6	4.3	
**Foramen** **Infraorbitale**	**LIOW**	**LIOL**	**RIOW**	**RIOL**	**LIOW**	**LIOL**	**RIOW**	**RIOL**	
Mean	2.7	**2.2 ^g^**	3.0	**2.5 ^h^**	2.9	**3.6 ^g^**	2.8	**3.5 ^h^**	**<0.05**
SD	±0.65	±0.50	±0.66	±0.55	±0.45	±0.62	±0.43	±0.56	
Min	2.1	1.6	2.1	1.8	2.2	2.5	2.2	2.5	
Max	4.1	3.2	4.0	3.5	3.7	4.5	3.7	4.8	

Abbreviation: LOW, left ovale width; LOL, left ovale length; ROW, right ovale width; ROL, right ovale length; LRW, left rotundum width; LRL, left rotundum length; RRW, right rotundum width; RRL, right rotundum length; LSOW, left supraorbitale width; LSOL, left supraorbitale length; RSOW, right supraorbitale width; RSOL, right supraorbitale length; LIOW, left infraorbitale width; LIOL, left infraorbitale length; RIOW, right infraorbitale width; RIOL, right infraorbitale length.

**Table 2:** The distances between the supraorbital, infraorbital foramen, impressio trigeminale, and median plane.

	TN Patients						Control Subjects						*p*-Value
	SO-MP		IO-MP		I TR-MP		SO-MP		IO-MP		I TR-MP		
	Right	Left	Right	Left	Right	Left	Right	Left	Right	Left	Right	Left	
Mean	24.6	25.1	24.9	24.9	26.5	26.3	25.9	26.2	24.3	25.4	26.8	26.7	>0.05
SD	±3.68	±2.99	±3.10	±2.44	±4.42	±3.43	±2.63	±3.09	±2.89	±3.34	±3.59	±3.67	
**Min**	17.7	22.1	20.2	19.7	20.4	20.6	21.0	21.1	20.9	19.6	21.8	20.7	
**Max**	32.8	32.1	31.2	29.8	38.2	37	28.9	32.7	30.3	32.7	36.8	35.7	

Abbreviation: SO-MP, supraorbital foramen to median plane distance; IO-MP, infraorbital foramen to median plane distance; I TR-MP, Impressio trigeminale to median plane distance.

**Table 3:** The distances between the superior orbital fissure, rotundum, and ovale foramen in trigeminal neuralgia patients and control subjects. The same letters indicate statistical significance which is less than 0.05.

	TN Patients						Control Subjects						*p*-Value
	FOS-FR		FR-FO		FOS-FO		FOS-FR		FR-FO		FOS-FO		
	Right	Left	Right	Left	Right	Left	Right	Left	Right	Left	Right	Left	
Mean	**6.4 ^a^**	**7.5 ^b^**	13.0	13.0	**20.4 ^c^**	**20.4 ^d^**	**5.4 ^a^**	**5.1 ^b^**	13.3	12.3	**18.8 ^c^**	**18.1 ^d^**	**<0.05**
SD	±1.61	±2.14	±2.0	±1.58	±1.85	±2.93	±1.33	±1.8	±1.97	±2.15	±1.98	±2.15	

Abbreviation: FOS-FR, fissura orbitalis superior to foramen rotundum distance; FR-FO, foramen rotundum to foramen ovale distance; FOS-FR, fissura orbitalis superior to foramen ovale distance.

**Table 4:** The distances between the superior orbital fissure, rotundum, and ovale foramen to the anterior, middle, and posterior clinoid processes in trigeminal neuralgia patients and control subjects. The same letters indicate statistical significance which is less than 0.05.

	TN Patients						Control Subjects						*p*-Value
	FOS-ANTC		FR-ANTC		FO-ANTC		FOS-ANTC		FR-ANTC		FO-ANTC		
	Right	Left	Right	Left	Right	Left	Right	Left	Right	Left	Right	Left	
Mean	17.0	16.9	20.5	19.6	23.6	24.1	16.6	16.0	19.9	18.5	24.9	23.6	>0.05
SD	±2.21	±1.67	±3.8	±2.56	±2.6	±2.37	±2.21	±2.09	±2.28	±2.24	±2.29	±2.02	
	**FOS-MEDC**		**FR-MEDC**		**FO-MEDC**		**FOS-MEDC**		**FR-MEDC**		**FO-MEDC**		
Mean	19.2	20.2	23.1	23.3	28.9	29.7	18.8	19.1	22.6	22.2	29.5	29.2	>0.05
SD	±2.61	±2.38	±3.38	±2.05	±2.09	±2.21	±2.24	±2.35	±2.63	±2.41	±2.26	±2.2	
	**FOS-POSTC**		**FR-POSTC**		**FO-POSTC**		**FOS-POSTC**		**FR-POSTC**		**FO-POSTC**		
Mean	**23.7 ^a^**	**23.9 ^b^**	**26.2 ^c^**	**25.4 ^d^**	26.9	27.0	**21.0 ^a^**	**20.9 ^b^**	**23.6 ^c^**	**22.9 ^d^**	26.6	26.2	**<0.05**
SD	±2.97	±1.96	±3.07	±2.67	±2.28	±2.18	±2.24	±2.58	±2.51	±2.84	±2.1	±2.62	

Abbreviation: FOS-ANTC, fissura orbitalis superior to anterior clinoid distance; FR-ANTC, foramen rotundum to anterior clinoid distance; FO-ANTC; foramen ovale to anterior clinoid distance; FOS-MEDC, fissura orbitalis superior to medial clinoid distance; FR-MEDC, foramen rotundum to medial clinoid distance; FO-MEDC; foramen ovale to medial clinoid distance; FOS-POSTC, fissura orbitalis superior to posterior clinoid distance; FR-POSTC, foramen rotundum to posterior clinoid distance; FO-POSTC; foramen ovale to posterior clinoid distance.

## Data Availability

The data retrieved from the author’s own research and university archive used for measurements in this study are not publicly available due to privacy restrictions.

## References

[1] Monkhouse S., Monkhouse S. (2006). The trigeminal nerve. Cranial Nerves Functional Anatomy.

[2] Binder D.K., Sonne D.C., Fischbein N.J., Binder D.K., Sonne D.C., Fischbein N.J. (2010). Trigeminal nerve. Cranial Nerves: Anatomy, Pathology, Imaging.

[3] Sabancı P.A., Batay F., Civelek E., Al Mefty O., Husain M., Abdulrauf S.I., Karasu A. (2011). Meckel’s cave. World Neurosurg..

[4] Malhotra A., Tu L., Kalra V.B., Wu X., Mian A., Mangla R., Michaelides E., Sanelli P., Gandhi D. (2018). Neuroimaging of Meckel’s cave in normal and disease conditions. Insights Imaging.

[5] Bindra A., Rath G.P. (2019). Etiopathogenesis of trigeminal neuralgia. Handbook of Trigeminal Neuralgia.

[6] Neto H.S., Camilli J.A., Marques M.J. (2005). Trigeminal neuralgia is caused by maxillary and mandibular nerve entrapment: Greater incidence of right-sided facial symptoms is due to the foramen rotundum and foramen ovale being narrower on the right side of the cranium. Med. Hypotheses.

[7] Graff-Radford S., Gordon R., Ganal J., Tetradis S. (2015). Trigeminal neuralgia, and facial pain imaging. Curr. Pain Headache Rep..

[8] Stiles M.A., Mitrirattanakul S., Evans J.J. (2007). Clinical Manual of Trigeminal Neuralgia.

[9] Kaplan M., Erol F.S., Ozveren M.F., Topsakal C., Sam B., Tekdemir I. (2007). Review of complications due to foramen ovale puncture. J. Clin. Neurosci..

[10] Oz U., Orhan K., Aksoy S., Ciftci F., Özdoğanoğlu T., Rasmussen F. (2016). Association between pterygoid hamulus length and apnea hypopnea index in patients with obstructive sleep apnea: A combined three-dimensional cone beam computed tomography and polysomnographic study. Oral Surg. Oral Med. Oral Pathol. Oral Radiol..

[11] Lamsal L., Rath G.P., Rath G.P. (2019). Introduction to trigeminal neuralgia. Handbook of Trigeminal Neuralgia.

[12] Umamaheshwara Rao W., Josh M., Rath G.P. (2019). Therapeutic Outcome and Future Scopes in the Management of Trigeminal Neuralgia. Handbook of Trigeminal Neuralgia.

[13] Bangash T.H. (2011). Trigeminal neuralgia: Frequency of occurrence in different nerve branches. Anesthesiol. Pain Med..

[14] Capel C., Peltier J. (2012). Commentary on Trigeminal Neuralgia: Frequency of Occurrence in Different Nerve Branches. Anesthesiol. Pain Med..

[15] Erbagci H., Kizilkan N., Sirikci A., Yigiter R., Aksamoglu M. (2010). Computed tomography based measurement of the dimensions of foramen ovale and rotundum in trigeminal neuralgia. Neurosciences.

[16] Liu P., Zhong W., Liao C., Liu M., Zhang W. (2016). Narrow Foramen Ovale and Rotundum: A Role in the Etiology of Trigeminal Neuralgia. J. Craniofac. Surg..

[17] Gupta T. (2008). Localization of important facial foramina encountered in maxillo-facial surgery. Clin. Anat..

[18] Apinhasmit W., Chompoopong S., Methathrathip D., Sansuk R., Phetphunphiphat W. (2006). Supraorbital Notch/Foramen, Infraorbital Foramen, and Mental Foramen in Thais: Anthropometric measurements and surgical relevance. J. Med. Assoc. Thai.

[19] Ashwini L.S., Mohandas Rao K.G., Saran S., Somayaji S.N. (2012). Morphological and morphometric analysis of supraorbital foramen and supraorbital notch: A study on dry human skulls. Oman Med. J..

[20] Nanayakkara D., Peiris R., Mannapperuma N., Vadysinghe A. (2016). Morphometric Analysis of the Infraorbital Foramen: The Clinical Relevance. Anat. Res. Int..

[21] Orhan K., Misirli M., Aksoy S., Seki U., Hincal E., Ormeci T., Arslan A. (2016). Morphometric analysis of the infraorbital foramen, canal, and groove using cone beam CT: Considerations for creating artificial organs. Int. J. Artif. Organs.

